# Supplementary material for: Modeling and prediction of the 2019 coronavirus disease spreading in China incorporating human migration data
Source: PLoS One. 2020 Oct 27;15(10):e0241171. doi: 10.1371/journal.pone.0241171 (PMC7591076; doi:10.1371/journal.pone.0241171)
Supplement: Attachment — Submitted filename: Author_reply_20200722.pdf [file pone.0241171.s001.pdf]

Authors' Responses to Editor's and Reviewers' Comments on
"Modelling and Prediction of the 2019 Coronavirus Disease Spreading in China Incorporating Human Migration Data"

We would like to thank the Editor and all reviewers for their helpful comments and suggestions, which have been taken into consideration in the revision of this paper.

The requirements pointed out by the editor have been addressed. Our specific responses are:

1. The paper is formatted in LaTeX using the PLoSONE template.
2. We have added the links in the data availability statement for the Baidu data and the infection data from the National Health Commission of China.
3. We have removed the Baidu map from Fig. 3 to avoid possible copyright issues.
4. We have used the PLoSONE template.

Reviewer: 1

Comments to the Author

Please use the space provided to explain your answers to the questions above. You may also include additional comments for the author, including concerns about dual publication, research ethics, or publication ethics. (Please upload your review as an attachment if it exceeds 20,000 characters)

Reviewer #1: This study used SEIR compartments to simulate the dynamics of COVID-19. It is an important issue now but there are some concerns as follows.

1. Many studies incorporated migration data into SEIR model for simulating epidemic dynamics. Authors need to highlight the significant findings of the study.

Authors' Response: This work was completed on February 19, 2020 (medRxiv 10.1101/2020.02.18.20024570). We used a short historical epidemic spreading data and migration data to develop the model and the corresponding system identification algorithm. At the time of performing this work, there was no attempt in combining SEIR model, migration data and system identification techniques to analyze and predict the spread of COVID-19. The results thus have important indicative values on the effectiveness of using limited initial outbreak data in predicting pandemic progression. Remarks have been added to the Discussions section to highlight this. The main findings were listed in the Results section.

2. Recent related studies on modeling COVID-19 which simulating the dynamics Wuhan and Hubei for estimated infected persons were published. Authors need to add more comparisons with these studies.

Authors' Response: The following information has been added to the Results section.

"For Wuhan, our model shows that the cumulative number of infections was 105,244 (95% CrI [64297,146191]), which was consistent with previous estimation of 75,815 infected cases (95% CrI [37304,130330]) [15]".

[16] Wu JT, Leung K, Leung GM. Nowcasting and forecasting the potential domestic and international spread of the 2019-nCoV outbreak originating in Wuhan, China: a modelling study. The Lancet. 2020 Feb 29; 395(10225):689-97.

3. Some notations need to be further clarify. For example, the notation of denominators in Equation 6 and Equation 7 should be P_i (t) and P_j (t), respectively.
Authors' Response: The notations have been revised.

4. If N_i^s (t) means the susceptible population in city i at time t, isn't it similar to S_i (t)? please explain their difference more clearly.
Authors' Response: Thank you for pointing this out. N_i^s represents the size of the group of susceptible, infected, exposed and removed individuals. Thus, we have N_is(t_0) = S(t_0)+E(t_0)+I(t_0)+R(t_0). This has been included in the Method section of revised paper.

5. Which is your notation of initial infection number? I_i (t_0) in line 228 or λ_I I_i (t_0) in line 236?
Authors' Response: I_i (t_0) represents the actual infected number at time t_0, while λ_I I_i (t_0) represents the initial infection number used in the model. We have clarified this in the paper.

6. What is the fitting result of the following parameters: δ_i, λ_I, and λ_E?
Authors' Response: The fitting result of δ_i, λ_I, and λ_E have been added, while Figure 6 (c) shows the distribution of \delta_i.

7. Figure 5 displays the result of forecasting; it should add 95% confidence intervals or error bars to show the variations of estimated values.
Authors' Response: The 95% confidence intervals (CrI) have been added to Figures 4 and 5, and in the text.

8. What is the spatial variation of the prediction? For example, whether the cities strongly interacting with Wuhan have more precise prediction results than the other cities? Or, whether high-population-density cities have more accurate predictions? These comparisons may reflect the value of incorporating human migration data into a SEIR model so that model results can benefit real epidemic prevention tasks.
Authors' Response: The experimental results show that several factors, such as strong interaction with Wuhan and high population density, influence the prediction results to some extent. Actually, the spread of COVID-19 in a city is highly influenced by the control measures, which vary from city to city. If a city adopted strict control measures, the spread of COVID-19 may be much slower and less severe than the predicted results.

Reviewer: 2
Comments to the Author
This is a sound analysis of two publicly available data set focusing on intercity migration in China.
The authors may benefit from a recent paper on migration and covid-19 spread published in April issue of Migration Letters journal. That can be useful to better frame the context of this paper indicating wider link between human mobility and disease diffusion.
Authors' Response: This work was completed on February 19, 2020 (medRxiv 10.1101/2020.02.18.20024570). We used a short historical epidemic spreading data and migration data to develop the model and the corresponding system identification algorithm. At the time of performing this work, there was no attempt in combining SEIR model, migration data and system identification techniques to analyze and predict the spread of COVID-19. The results thus have important indicative values on the effectiveness of using limited initial outbreak data in predicting pandemic progression. Remarks have been added to the Discussions section to highlight this.

Authors may revisit the sentence in second page: "The COVID-19 outbreak, however, began to occur and escalate in a special holiday period in China (about days surrounding the Lunar New Year), during which a huge volume of intercity travel took place, resulting in outbreaks in multiple regions connected by an active transportation network." Because now we know the virus was out and about in as early as early November.
Authors' Response: We have checked the literature and available data carefully, and found that the "official" data (up to today from the Chinese National Health Committee) indicated the earliest confirmed case in China being December 8, 2019. Indeed, the spread could have started earlier, but our data analysis could only work according to the official data which showed surges in infected numbers in many Chinese cities beginning mid January, which was the period of "spring rush" in China. We have also edited the text so as to emphasize that we referred to the rapid spread in China which was in the period before the Lunar New Year when huge volume of intercity travel took place.

The data needs to be critically presented; Authors indicate the possibility of incompleteness or inaccuracy of official covid data but it seems they assume Baidu data is free of problems. It is important to note the selectivity bias here. This data is collected by an app, which means there are a lot of questions about its representability. This should be clearly noted so readers can interpret it accordingly.
Authors' Response: Several works adopted Baidu data to investigate the spread of COVID, which have been cited in the paper. Also, we clarified that the Baidu data were

expected to be inexact and served to provide indicative travel volumes which were sufficient for the model fitting. This would serve to alert our readers about this issue.

[14] Chinazzi M, Davis JT, Ajelli M, Gioannini C, Litvinova M, Merler S, y Piontti AP, Mu K, Rossi L, Sun K, Viboud C. The effect of travel restrictions on the spread of the 2019 novel coronavirus (COVID-19) outbreak. Science. 2020 Apr 24; 368(6489): 395-400.
[15] Lai S, Ruktanonchai NW, Zhou L et al. Effect of non-pharmaceutical interventions to contain COVID-19 in China [published online May 4, 2020]. Nature. 2020;10.1038/s41586-020-2293-x. doi:10.1038/s41586-020-2293-x

In the conclusion, authors state "The Coronavirus Disease 2019 (COVID-19) epidemic has hit China hard, 331 and as of February 20, 2020, a total of 74,579 infection cases have been 332 confirmed in China, with death toll reaching 2,119." It is a live incidence but it can be useful if they can include the latest statistics regarding the pandemic while making sure the data and analysis refer to an earlier period.
Authors’ Response: We have revised the Introduction to include the latest worldwide figures while emphasizing that this work was completed on February 20, 2020.
